# Supplementary material for: Complexity reduction by symmetry: Uncovering the minimal regulatory network for logical computation in bacteria
Source: PLoS Comput Biol. 2025 Apr 24;21(4):e1013005. doi: 10.1371/journal.pcbi.1013005 (PMC12048163; doi:10.1371/journal.pcbi.1013005)
Supplement: S1 text — Supplemental information and formal definitions; detailed methodology breakdown; supporting results; algorithms explanation and pseudocode. (PDF) [file pcbi.1013005.s001.pdf]

Supporting Information

Complexity reduction by symmetry: uncovering the minimal regulatory network for logical computation in bacteria

Luis A. Álvarez-García[1‡], Wolfram Liebermeister[2], Ian Leifer[1], Hernán A. Makse[1*]

1 Levich Institute and Physics Department, City College of New York, New York, NY 10031, USA
2 Université Paris-Saclay, INRAE, MaIAGE, 78350 Jouy-en-Josas, France

‡luisalvarez.10.96@gmail.com * hmakse@ccny.cuny.edu

A Specifics of gene regulation dynamics

Gene expression dynamics in the GRN can be modeled using ordinary differential equations (ODEs) [1, 2] for mRNA and protein concentrations, assuming known gene-regulatory functions [3].

While we will not model signal transmission dynamics, it is necessary to understand how a network of regulatory arrows can be translated into a dynamical system and vice-versa. This clarifies both the importance of network structure and the importance of other, quantitative and gene-specific details that are not represented by network structure alone. In a simple lumped model, each gene is described by a single dimensionless expression value, representing the protein concentration, that is, the gene product. We consider a protein with concentration x_i, encoded by gene i and regulated by a set of genes j expressing transcription factors with activities y_j. The expression dynamics of this model can be modeled by the following lumped ODE:

$$\frac{dx_i(t)}{dt} = -\alpha x_i + F[\gamma_j f_K(y_j)], \tag{1}$$

where $x_i(t)$ is the time-dependent protein concentration expressed by gene i, α corresponds to its degradation constant (including the often dominant effect of dilution [4]), γ_j is the maximum synthesis rate of the protein product and $f_K(y_j)$ is the interaction or input function between TF y_j and the binding site of gene i which depends on the dissociation constant K between them.

The coupling term $f_K(y_j)$ for an individual TF y_j interaction is usually modeled as a sigmoid function such as a Hill function [1,2], or a Heaviside step function, its Boolean logic approximation [5,6]. Qualitatively, the Heaviside step function can be thought of as a Boolean logic function in the following intuitive manner [6]: each gene can take the "on" or "off" state, an activation signal being a proxy for *"turning on"* the gene while an inhibition signal is a proxy for *"turning off"* the gene.

The function $F[\cdot]$ combines the regulatory input functions of all the inputs y_j of the gene i. They have been proposed and measured in Refs. [7, 8]. When the input function combines the activity of several TFs, the input functions can be taken as different logic gates. For example, a logic AND gate with $F[\cdot]$ being a multiplicative function of its individual gene input, or OR gates with $F[\cdot]$ being additive on the input [9]. In general, the fact that the input function collects the activity of more than two TFs, implies that the biological graph is actually a hypergraph, with each input function defining a hyper-edge. These input functions can be more complicated forms that involve many-body interactions where three or more inputs interact in a common way.

While these interactions are treated by hypergraphs, not by graphs, in this paper we consider only two-body interactions captured by a graph, since most of the results are not affected by this difference, as long as the hyper-edge is the same for all interactions. We leave the study of hypergraphs for a follow-up study, yet these considerations do not affect the general conclusions about the symmetries of the GRNs.

Equation (1) contains enormous simplifications, since, in reality, one would need to know the precise values of all parameters that define the model to make an exact model. These effective parameters capture everything from transcription and translation rates, protein folding, to binding and unbinding of the TF to DNA, not

to mention ribosome and polymerase binding, as well as the degradation lifetimes of mRNA and proteins [4]. This set of events are lumped into one single edge representing the whole process in the GRN. This large set of parameters are mostly unknown to the modeler trying to understand the regulatory process, therefore the parameters used in 1 are lumped parameters to account for these phenomena effectively [1].

Crucially, in order to do a precise prediction for the entire GRN one would need to know these parameters for every edge of the network, in our case for our *E. coli*'s GRN-operon network there are 1,835 edges, for example. It would be pretty much impossible to model this in an exact manner using current knowledge of biology. What we do instead is to take the most drastic approximation and take the parameters to be the same for every edge [10]. This implies that we will search for the highest possible symmetry state of the network. Any deviation from this approximation will incur in some network symmetry breaking by heterogeneity of parameters. Then, the question remains whether this symmetry breaking is strong enough to break the synchronized dynamical patterns of gene expression observed experimentally. In fact, this approximation can be relaxed to have the same edge parameters for the outputs of genes involved in a fiber, but the parameters could still be different for different genes in different fibers. Importantly, because the purpose of this work is to study the overall structure to determine how it impacts the information flow and decision-making process, general conclusions about the structure of the minimal GRN, the presence of logic circuits and the interactions between them still hold even if the synchronizations are reduced to only correlations and the actual dynamics stemming from Eq. 1 would be more complicated.

The uniform parameter approximation allows us to do a large improvement in understanding the structure of the network; as will be shown, it will reveal the ideal structure via symmetries from which small corrections due to the heterogeneous parameter space can be studied later on. The question that will arise then is whether the set of equations defined on the graph may break these symmetries or not. Our contention is that the heterogeneous parameters preserve the structure already imposed by the graph symmetries, and this is supported by the experimental evidence of gene coexpression patterns (synchronization) widely obtained for these systems [10].

B Graph fibrations and formal definitions

A (directed) *graph* $G = (N, E)$ is a pair of nodes N and edges E, where each edge $e \in E$ is an unordered (ordered) pair of nodes, i.e., $e = (i, j)$ for $i, j \in N$ [12]. It is customary to define two functions $s, t : E \to N$ that map each the edge to its respective source and target nodes: $s(e) = s(i, j) = i$ and $t(e) = t(i, j) = j$. A *sub*graph is a graph $g = (n, e)$ where $n \subseteq$ and $e \subseteq E$. An *induced* subgraph, in turn, is the subgraph resulting from taking a subset of nodes of the original graph and *all* the edges between them.

A directed graph is strongly connected if there is a path between all pairs of vertices, in both directions. A *Strongly connected component* (SCC) of a directed graph is a maximal induced subgraph that is also strongly connected.

A *graph morphism* $\varphi : G \to B$ is a mapping between two graphs $G = (N_G, E_G)$ and $B = (N_B, E_B)$ given by two functions mapping the nodes and edges respectively, $\varphi_N : N_G \to N_B$ and $\varphi_E : E_G \to E_B$, that satisfy $s_B(\varphi_E(e)) = \varphi_N(s_G(e))$ and $t_B(\varphi_E(e)) = \varphi_N(t_G(e))$ for every edge $e \in N_G$ [12], a sort of commutative relation.

Simply speaking, if two nodes are connected in G, they are connected in B in such a way that the incidence relationship between the source node and the target node is preserved for their respective images. This means that the edges are mapped so that the edge image connects the image of the source node to the image of the target node.

A fibration has a stronger conservation of the graph's structure than a plain morphism, since it not only preserves the incidence relation but also requires the lifting property, which preserves the input trees of all image nodes in the base.

In order to determine the fibers we start by defining the *input set* I_i of node i as the set of incoming edges $e \in E_G$ such that $t(e) = i$ along with their respective sources $j = s(e)$. The *input tree* T_i of node i is the

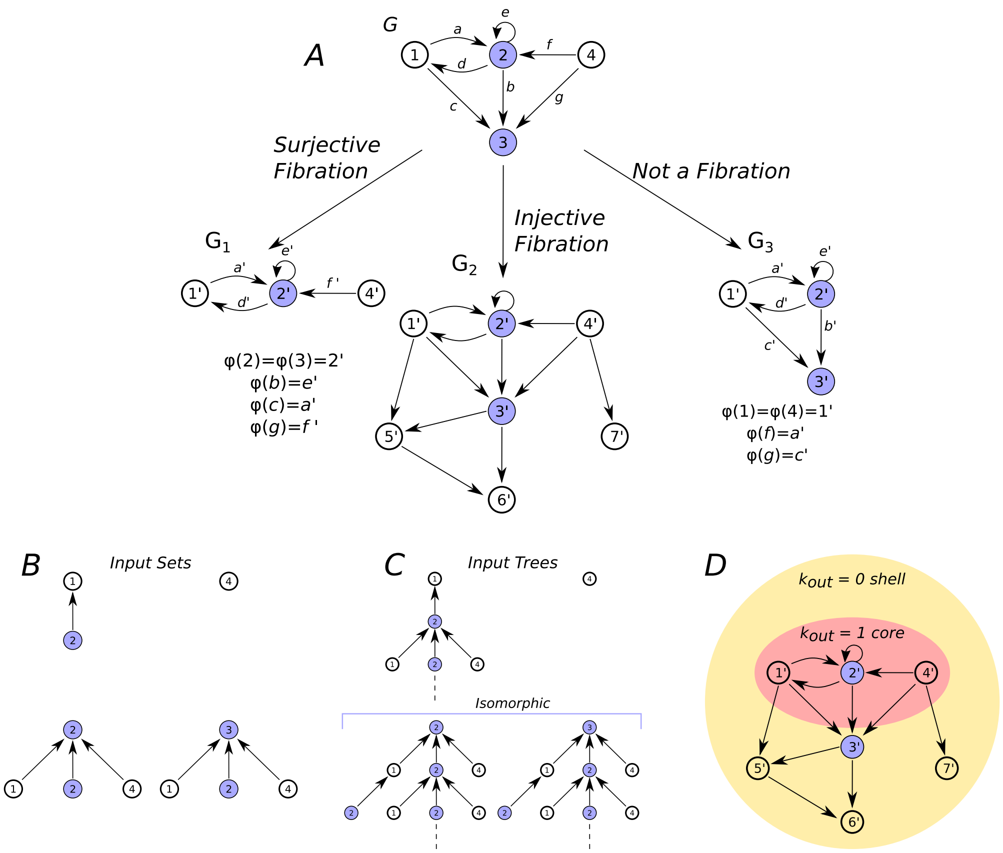

Fig. A. Fibration and k_{out}-core decomposition. **A** Graph G, a subgraph of the GRN of *E. coli*, shows a Fibonacci building block with class number $|\varphi = 1.6180, \ell = 2\rangle$ [10,11]. All three mappings are morphisms since the images of all the nodes in G_1, G_2 and G_3 are connected only when corresponding nodes in G are connected, respecting the incidences. The mapping $G \to G_1$, in the left, corresponds to a surjective fibration: all nodes with isomorphic input trees are collapsed to one (nodes 2 and 3 collapsed to $2'$), all input trees are preserved, hence the lifting property is satisfied. Mapping $G \to G_2$ is an injective fibration. Indeed, it is easy to see that the original graph is embedded in G_2 making this map a morphism where all input trees are preserved. Some nodes and edges are added but without breaking the original input trees. The mapping $G \to G_3$, which maps node 4 to $1'$ does not correspond to a fibration given that the input-tree of node 4 (seen on **B**) is not preserved in its image node $1'$ in graph G_3, the same problem occurs with the images of nodes 2 and 3 ($2'$ and $3'$ respectively), their input trees are not preserved as the former input from node 4 is lost. Edges a' and c' cannot be uniquely lifted at $\varphi(2)$, since they need to be lifted to a, f and c, g, respectively, for the mapping to be a morphism. In practical terms, since the input from node 4 is lost, graph G_3 represents an entirely different dynamical system from graph G. If the graph G represents a GRN, genes $2'$ and $3'$ in G_3 would have a different expression pattern than genes 2 and 3. **B** Shows the input sets and **C** the input trees of nodes in graph G. The input set of node 2 is repeatedly attached to node 2 in every layer of the trees, due to its self-loop, this process is repeated ad infinitum. As a result, the input trees of nodes 1, 2 and 3 are infinite; however, since G has only 4 nodes, it suffices to verify the isomorphism up to the third layer of their trees, hence nodes 2 and 3 are determined to have isomorphic input trees. **E** Example of the k-core decomposition of graph G_2 from **A**. Even though node $5'$ on the outer $k_{\text{out}} = 0$ shell (in red) does have one output, once nodes $6'$ and $7'$ in the shell are removed, it will then be left with no output and will be removed as well. All the remaining nodes in the $k_{\text{out}} = 1$ core have at least 1 output after doing this process.

input sets of the input sets taken recursively. Input trees can be finite or infinite depending on the existence of the cycles in the network. An infinite input tree occurs anytime a node receives signals from a strongly connected component of one (an autoregulation loop) or more nodes.

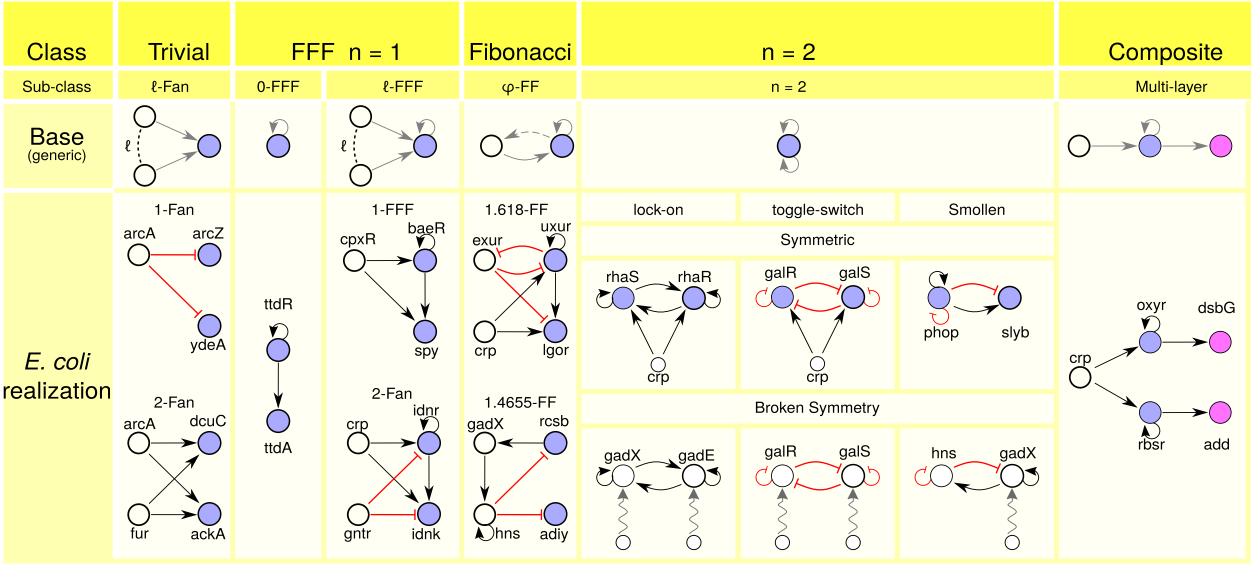

Fig. B. Canonical fiber building blocks. These correspond to the canincal fiber building blocks observed in the GRNs of *E. coli* and *B. subtilis*, with examples taken from *E. coli*. The networks can be seen as assemblies of 5 basic classes of fibration building blocks: (i) Trivial fibers. A number ℓ of external regulators identically regulate the genes in a fiber, which then show synchronous dynamics. Operons with only one promoter belong to this class, where colored nodes represent genes belonging to the operon (perhaps with more colored nodes in the fibers, depending on the number of genes in the operon). (ii) The feedforward fiber and its sub-classes of ℓ-FFF with ℓ external regulators. The FF fiber is defined by a feedforward motif with a self-loop in the synchronous set of genes, and the number of ℓ external regulators. (iii) The Fibonacci fiber, φ-FF. A more complex building block, defined by a fractal dimension branching ratio that occurs given the presence of a self-loop and a feedback regulation from the fiber back to the regulator(s). The Fibonacci fibers observed in *E. coli* have a branching ratio between 1 and 2, placing this building block in between the FFF fibers and the n=2 fibers. (iv) The n=2 fibers, defined by two self-loops in the synchronized genes. When this symmetry is broken it forms the memory and oscillatory logic circuits embedded in the SCCs. And finally (v) composite fibers of the previous ones. By adding different types of the previous 4 building blocks, in a sequential manner, a composite fiber is obtained. An interesting consequence of this is the synchronization of genes that may be far apart from each other and don't share any regulation.

A *graph isomorphism* denotes a graph morphism whose inverse is also a morphism, i.e. a graph isomorphism is a bijective (one-to-one correspondence) graph morphism. Formally, a graph morphism $\tau : G \to C$, between graphs $G = (N_G, E_G)$ and $C = (N_C, E_C)$, is a graph *isomorphism* if and only if for every edge $(i, j) \in E_G$ there is an edge $(\tau(i), \tau(j)) \in E_C$.

Two graphs are said to be isomorphic if there exists an isomorphism between them. Fig AB demonstrates an example of two isomorphic input trees. Intuitively, the topology of these trees is exactly the same, meaning that the graphs are the same except for a relabeling of the nodes (and also the edges), and the isomorphism condition above is satisfied.

An input tree graph isomorphism defines an equivalence relationship between nodes in the graph where the equivalence classes are *fibers:* nodes with isomorphic input trees.

That is, the fibration collapsing nodes in a fiber into a single node in the base conserves the dynamics of the graph. Thus, in terms of an admissible set of equations attached to the graph, fibers lead to the existence of synchronous solutions for the nodes within the fibers. This is called *cluster synchronization,* in this case corresponding to gene coexpression patterns [10, 11, 13].

We have shown before [5, 10, 11, 14] that the use of fibrations allows for the breakdown of the network into its fundamental synchronized building blocks, the *fiber building blocks.* Each fiber belongs to a fiber building

block, defined as the induced subgraph formed from the nodes in the fiber, and the nodes that send inputs to the fiber (the regulators). In the case where the fibers send signals back to any of its regulators (i.e. there is signal feedback from the fiber to its regulators), all nodes that belong to the shortest path from the fiber to the regulators must also be included.

We find that these fiber building blocks can be precisely characterized by just two numbers n (or φ) and ℓ, defining the $|n, \ell\rangle$ classes [11]. Here ℓ corresponds to the number of genes (externally) regulating the fiber, the external regulators, and n the number of cycles within the fiber. n can be of integer or fractal dimension (represented by φ in these cases), depicting if the input tree's size growth can be described by an integer number or not. The latter being the case when there is a cycle between the fiber and the regulators.

The full list of fibers and their classification in the *E. coli* GRN can be seen in the Supplementary Information File from [11]. For *E. coli* and *B. subtilis*, all the different fiber building block structures are classified by just 5 basic canonical structures shown in Fig B, which implies a nice reduction in complexity of these fundamental structures.

B.1 Gene coexpression and fiber building blocks.

In GRNs, a symmetry fibration describes the synchronized expression of genes with isomorphic input trees or, biologically, gene co-expression [5, 10]. As discussed, the underlying uniformity assumption is that the regulatory input functions of the genes, as well as their parameters, are identical between all genes in a fiber. In biological reality, this is clearly not true: different genes (even with the same input edges) will show different input functions, mRNA lifetimes, etc, and so genes in a fiber will not show strict synchronicity. However, we assume that these genes will still show correlated dynamics and that deviations from strict synchronicity can be described as a weak departure from the exact synchronous state [10]. Thus, we consider a simplified picture of GRNs in which this symmetry assumption for gene regulation functions holds.

Synchronization requires that isomorphic input trees do not experience any significant communication delays which would cause asyncronicity and that the constants involved in Eq. (1) are approximately the same for all the genes in a fiber, as discussed. This is not difficult to satisfy in GRNs where interactions depend mainly on the TFs and not so much on the binding site of the regulated genes. Small variations resulting from mismatching parameters appear to result in weak symmetry breaking, creating a slight reduction in the synchronization and correlation of expression levels within the fibers [10].

The co-expression patterns obtained here are more general than traditional co-expression patterns in operons and regulons. Indeed, all genes in an operon with a single common promoter are in the same fiber. Such operons can be thought of as examples of *"trivial"* fibers, see Fig B. The same applies to nodes that belong to only one regulon and share the same regulatory TF, they form a *"trivial"* fiber since they are only regulated by one gene in an identical manner. Generally speaking, symmetry fibrations allow us to find not only these trivial fibers but also broader co-expression patterns among genes that are far away in the genome and more complex patterns of synchronization. Particularly interesting fibers, shown in Fig B, are the Fibonacci fibers where the presence of a feedback loop between the fiber and its regulator (forming a SCC), plus a self-loop in the fiber produce an input tree with a branching ratio of fractal dimension, as seen in Fig B. Other complex fibers are composite multi-layered fibers, in which nodes that are not regulated by the same node are still synchronized because their regulators belong to a fiber.

It is important to note that the theory of fibrations only predicts the existence of these symmetric synchronized solutions. But not all synchronous solutions must be symmetric. More importantly, symmetries do not guarantee that the synchronous solution will be stable, and solutions may (in theory) be dynamically unstable [15, 16].

Thus, fibrations do not cover all dynamics in the original network, and guarantee existence but not stability. Fibrations cannot guarantee that these symmetric solutions are actually relevant dynamic attractors and not unstable. The stability of the synchronous solution needs to be studied a posteriori, and it depends on the particular type of ODE used to describe the dynamics. Thus, different stabilities can be obtained for different models, whether we use a Hill function or a linear interaction term or a step function, for instance, in the ODE. In fact, there are very interesting bifurcations that can exist for a given dynamical model,

and bifurcation can be symmetry preserving or symmetry breaking. Each fiber needs to be investigated separately for each model. The stability and bifurcation analysis of the circuits found in bacterial GRNs are investigated in detail in Ref. [17].

In summary, by studying the input trees of all nodes in a graph we can determine all the symmetries of the network in terms of signal processing. Specifically, the symmetries of a network are given by the equivalence relations induced by input tree isomorphisms. Therefore, symmetric paths can be removed while preserving the flow of signals, which is concretely related to the concept of fibration [13, 18] and its implications for dynamics in biological networks [5, 11].

C The ComSym analysis step by step

C.1 Step I: The symmetry fibration – Collapsing a graph into its minimal base by surjective fibrations

The main reduction process is the application of surjective minimal fibrations, or *symmetry fibrations*, which reduces the original network to its minimal base. This is the fibration that collects the maximal symmetry. We first identify all isomorphic input trees (see SI and Refs. [11,19] for a discussion on algorithms), obtaining the least amount of fibers, or colors, hence also receiving the name minima balanced coloring. Afterwards, all fibers can be collapsed into a single representative node, obtaining as a result the minimal base. This base network represents the reduced effective model network for the original system and has the same original signaling flow but with no redundant pathways. Since nodes in the same fiber share identical inputs, these are not changed; however, they generally have different outputs. When the collapsing is done, all previous outputs from all nodes in the fiber must be "rewired" so that their new source node is now the collapsed fiber-node.

DeVille and Lerman [13] have shown that any surjective fibration $\varphi : G \to B$ induces synchronization on nodes i and $j \in N_G$ if $\varphi(i) = \varphi(j) \in N_B$. Thus, for the case of a symmetry fibration, all nodes within the same fiber are synchronous. This guarantees that the dynamics for both the original network and the base network are the same, all the collapsed nodes dynamics are identical to the representative node they were collapsed into.

Importantly, this reduction is valid for any signal-processing network when a substantial reduction in network size is desired without losing signaling flow [11].

C.2 Step II: Injective Fibrations

An injective fibration can help us formalize the intuitive notion that under certain conditions the dynamics of an entire system may be driven by only a subset of its constitutive elements. This is formalized by *Lemma 5.2.1* in DeVille and Lerman, Ref. [13], where it is shown that for an injective (one-to-one) fibration $\varphi : G \to G_2$ the dynamics of the bigger graph G_2 is driven by the dynamics of the smaller graph G. In fact G is a subgraph of G_2. This can be seen on Fig A with the injective fibration from G to G_2.

The emphasis here is on the injective nature of the map φ. For a mapping to a larger graph to be a fibration, like the one shown in Fig A, it must satisfy the lifting property. This requires that all edges in G_2 whose target node is an image from a node in G (i.e. $1', 2', 3', 4'$), can be uniquely *lifted* to an edge in G. This implies that no new edges are allowed to target any of the nodes of the original graph (that is, $1', 2', 3', 4'$), satisfying the lifting property. As a consequence, all the added nodes in G_2 (i.e. $5', 6', 7'$) must strictly be only targets of the image nodes. Hence, signals flow only outward from G, and therefore the dynamical state of the outer nodes is driven by the dynamics of the original smaller graph G. In other words, the subgraph G of G_2 drives or controls the entire dynamics of G_2. This, in turn, guarantees that the dynamics of the original graph is preserved in the image graph G_2.

However, the issue for us is how to reverse this process to obtain the driver subgraph G given G_2. We

propose that the k-core decomposition of a graph is the tool that allows us to perform the *inverse* injective fibration to find the driver subgraph of the network G. This will allow us to distinguish between the nodes that shape the network dynamics and those that are just being driven.

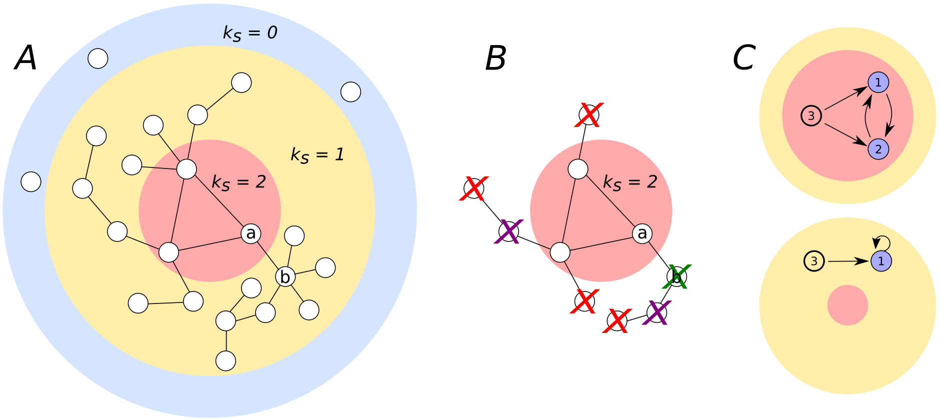

Fig. C. k-core decomposition. **A** Schematic drawing of the k-core decomposition of an undirected network. Even though node b has a higher degree than node a, it is connected to nodes with smaller degree and has therefore a smaller coreness than node a. **B** Example of how to obtain the $k = 2$ core of the network in **A**. First, the nodes with degree less than 2 are removed. The remaining nodes are the ones shown, successive iterations remove the nodes crossed with colored x. Different colors stand for successive iterations: red is the first iteration, the second one is purple and the last one green. **C** The bottom network corresponds to the symmetry fibration of the network on top. The SCC of nodes 1 and 2 is also a fiber, after collapsing the fiber, the SCC is lost, becoming a single node with a self-loop. The collapsed network then becomes an acyclic graph, without a $k_{\text{out}} = 1$ core.

The k-core decomposition reveals the key nodes that drive the network's dynamics Formally, the k-core of a network is the maximal induced subgraph, where the degree of each node within the k-core is at least k. It consists of "peeling off" layers, the k-shells of a network, by assigning the coreness index k_s to each node, corresponding to the respective shell they belongs to [20]. The k-shell corresponds to the set of nodes with coreness $k_s = k$. The coreness of a node is given by k if it belongs to the k-shell, that is, the k-core but not to the $(k + 1)$-core. The lower the coreness k_s of the node, the more peripheral it is [21], as can be seen in FigC.

For example, since nodes having a degree of at least 0 belong to the $k = 0$ core (all nodes), node b belongs to the $k = 0$ core and to the $k = 1$ core, as shown in Fig CA, but does not belong to the $k = 2$ core, so its coreness is $k_s = 1$.

Taking the k-core of a network corresponds to removing all nodes with degree less than k, calculate the new degrees in the new subgraph, and remove again nodes with degree less than k, iteratively until all remaining nodes have a degree of at least k. A node with coreness k has a degree of at least k, connecting to other nodes within the same core.

The coreness of a node captures the degree of the nodes to which it is connected. For example, node b in Fig CA has a smaller coreness ($k_s = 1$) than node a ($k_s = 2$), although b has a higher degree of 5 compared to the degree of a' of 3. This is because b is mostly connected to nodes with a degree of 1, in contrast to a which connects to nodes with a higher degree, making it more *"influential"* [21] or central.

To apply this concept to bacterial GRNs we must first extend it to directed graphs [20], where each node now is characterized by an *in-degree* and an *out-degree*, instead of the usual simple degree in undirected graphs. However, since we are interested in the direction of outward signaling flow, given that this case corresponds to the direction of regulation from one gene to another, we only need to consider the out-degree of each node. We are now interested in the k_{out}-core, which corresponds to the maximal induced subgraph where every node has at least an *out-degree* of k. This means that in this case we are iteratively removing nodes

with less *out-degree* than k. See for example in Fig AD the k_{out} core of network G.

In our case, we are only interested in genes that send any signals to other genes, and we want to remove the ones that do not. Hence, we want to remove the $k_{\text{out}} = 0$ shell of the network and obtain the $k_{\text{out}} = 1$ core. In a sense, we are trimming the frayed ends, or loose ends, of the network to unravel its core, this core network is the minimal GRN.

When collapsing only to the $k_{\text{out}} = 1$ core without applying the symmetry fibration, one also finds a subset of nodes that drive the entire graph. However, this may not necessarily yield the *minimal* subset, as there may be some redundancy in the signaling flow of the resulting network. In the case of a 2-node SCC whose nodes also belong to the same fiber, shown in Fig CC, given that both nodes 1 and 2 have an *out-degree* of one, they belong to the $k_{\text{out}} = 1$ core of the original network. However, when applying the symmetry fibration first, they collapse into a single node with a self-loop and no *out-degree*, which implies that they no longer are part of the $k_{\text{out}} = 1$ core.

Hence, as input tree isomorphisms give us the correct way to collapse a network while preserving signaling flow, the k-core decomposition, and in particular the $k_{\text{out}} = 1$ core, gives us the correct *inverse* fibration to reduce the network to its core network, its minimal set of nodes driving the dynamics.

C.3 Step III: Strongly Connected Components and their interactions

In undirected networks, the $k = 1$ core contains the giant connected components of the network, this can be seen in Fig CA. Analogously for directed networks, the $k_{\text{out}} = 1$ core is composed of the *strongly* connected components, the nodes that feed them signals and the connections between them. As shown in Fig AD the $k_{\text{out}} = 1$ core contains the SCC involving nodes 1 and 2 as well as node 4 that sends signals to it. As a corollary, all acyclic directed graphs have a null $k_{\text{out}} = 1$ core.

In this step, what we wish to find is how to break the minimal network into its components, i.e., how the SCCs interact with each other and what structure of the minimal network emerges from this. This will give us what we call the *large-scale structure of the minimal network*. After this, we "zoom in" onto the SCCs to analyze the *small-scale structures* inside of the SCCs in Steps IV and V.

C.4 Step IV: Broken symmetry circuits: hierarchy and identification

We find that circuits with broken fibration symmetry act as circuits performing basic logic computations in the GRN [5]. These computations are of two types: memory storage and timing via oscillations.

Gene duplication could explain the rich symmetries in GRNs Gene duplication is an important and major process in the evolution of a genome in which entire pieces of the chromosome are duplicated, resulting in the cell having two paralogue copies of a set of genes. This duplicates not only the gene but also its promoter region, the DNA sequence adjacent to the gene, where the binding sites to which the TF can attach are located.

In this way, gene duplication could work as a driver for fibration symmetries in transcription regulatory networks. By duplicating a gene in such a way that the paralogue genes share the same input relations, creates a fiber since the duplicated genes would have an isomorphic input tree. In this instance gene duplication works exactly as a lifting property, starting from the initial network and going into a new one with more symmetries and bigger fibers. Further mutations in the coding region then cause the two original copies of the gene to perform different functions, and thus diversify the bacteria's functions while creating bigger fibers.

Logic circuits are identified by starting with a symmetric circuit, which originates from a fiber building block, and breaking its symmetry by adding extra edges acting as regulators that break the symmetry in the fiber structure. Breaking of symmetry can be the result of a process of gene duplication that starts with the symmetric base of a fiber building block, which is duplicated to a (still) symmetric structure. This new

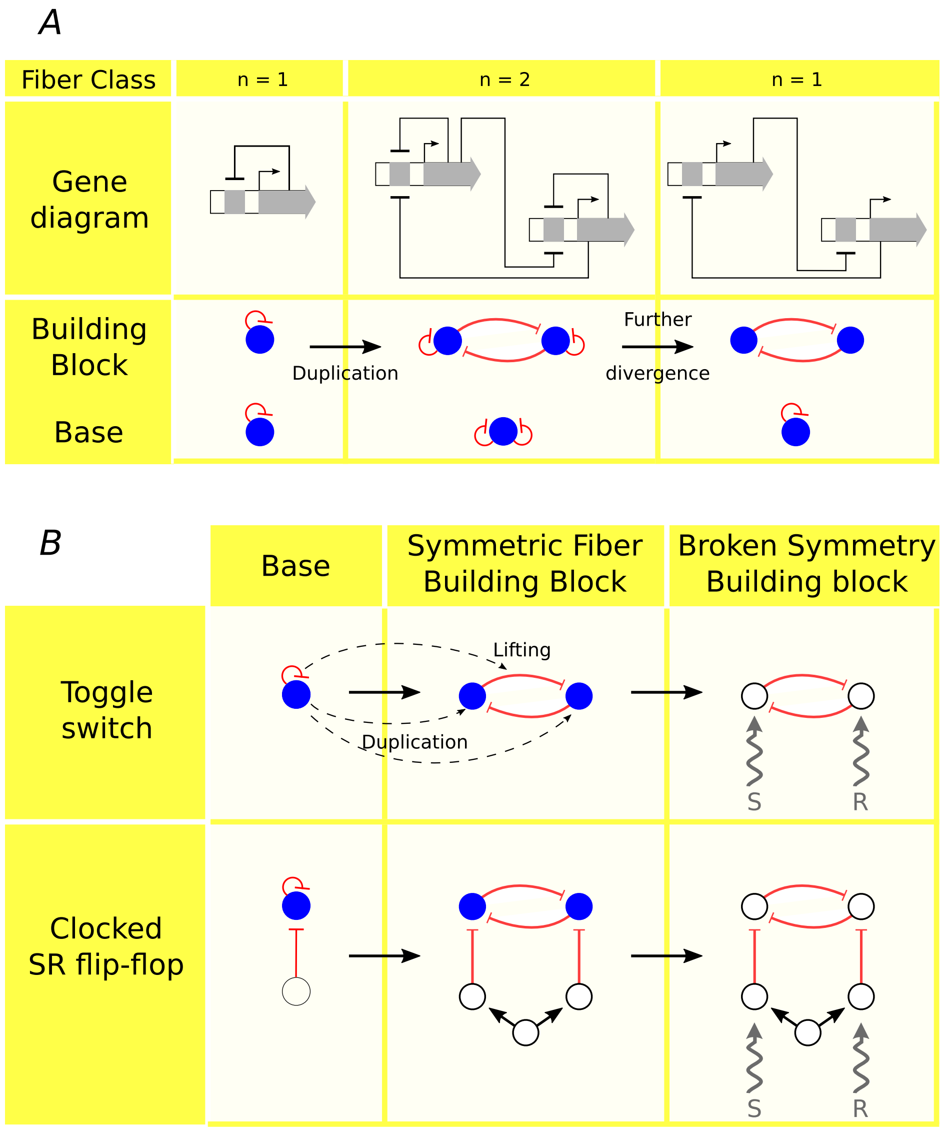

Fig. D. Broken symmetry circuits and duplication process. Starting with an initial simple structure a gene duplication process can create a symmetric fiber structure, analogous to a lifting process. **A** The structure resulting from the duplication process may need to undergo further modifications. **B** The circuit formed depends on the initial structure that is replicated, shown at the bottom. For both circuits, the symmetric form is shown on the center, while the broken symmetry process is shown on the right. The *set-reset (S-R)* inputs break the symmetry by sending different regulations to an otherwise synchronous pair of nodes. The *S-R* inputs can be any different nodes sending different signals or can be a same node sending different signals to the nodes in the fiber.

structure may then incorporate new regulations that result in the symmetry breaking on this duplicated circuit, as seen in Fig DB. Crucially, if the result of a duplication process respects the lifting property and preserves the fibration symmetry (see also Fig DA), this new obtained symmetric structure is prone to obtain new regulations that will then result in symmetry breaking.

Strictly speaking, the duplication process of a base does not necessarily produce a symmetric structure or a structure with the same fiber classification (see Fig DA). For example, if a self-inhibiting gene is duplicated into two genes, a precise duplication of the self-inhibition should produce a structure in which the two paralogue genes mutually repress each other as well as also self-inhibit themselves, see Fig DA. The original structure corresponds to a $n = 1$ FFF, while the new duplicated structure would in fact correspond to a $n = 2$

instead, which accounts for the two interactions that the new paralogue genes receive. However, the initially duplicated structure may undergo further divergent modifications, like the removal of the self-inhibitions in this case, that result in a structure that actually preserves the same fiber classification since the input relations are the same as in the original structure. Hence resulting in a structure with the same original dynamics that respects the lifting process. This new structure, when collapsed via a symmetry fibation gives as a result the actual original structure that got replicated. This further process of modifications after duplication can be thought of as a divergence process of the duplicated structure. In this case, there are now multiple synchronous genes where before there was only one.

The duplication process plus divergence *'opens up'* the base of a fiber building block, resulting in, at least, a pair of synchronous genes. In this instance, this process is identical to how the lifting property *'opens up'* a fiber from the base into the full set of synchronous genes. The identification of the liftying property as a duplication process could actually account for a possible explanation of the emergence of fibration symmetries in these GRNs and why biological networks present such a rich symmetric structure (see for example Fig 4 in [11].)

Symmetry breaking gives rise to gene-logic circuits Duplicating a self-inhibiting gene in such a way that the lifting property is respected implies that the duplicated circuit results in two mutually repressed (MR) genes forming a two-node negatively auto-regulated (NAR) fiber (a fiber that auto-regulates itself in an inhibitory manner) as the one shown in Fig DA. This duplicated circuit is still a symmetric circuit whose dynamics are not the same as the negative auto-regulated loop in the base, since each gene receives two inputs in the duplicated circuit, and the base receives only one. So, this duplication event does not conserve lifting, and the duplicating genes cannot be fiber to the base. If the genes further "lose" the autoregulations, then the resulting circuit is the lifting of the base, and the fibration can be applied back and the dynamics are conserved.

When different regulators are added to each gene, then the symmetry is broken, and the resulting circuit is a bistable switch known as a toggle-switch. This circuit corresponds to a structure analogous to a *flip-flop* in electronics [22] with the different inputs for each gene being the *'set' (S)* and *'reset' (R)* switches, see Fig DB. This circuit is the bistable toggle-switch [1, 5] that stores one bit of information given that as a bistable switch it has two possible stable and reciprocal states. One of these stable states correspond to one gene being expressed and the other one inhibited, and its reciprocal case being the other stable state. Even if the symmetry is restored and both inputs return back to being identical, the state of circuit remains unchanged, hence storing the previous state as one bit of information. It is possible to switch between the two states by toggling the *S-R* inputs to the circuit, hence why this is referred to as a bistable two-way switch.

The most simple form of a bistable switch correspond to this flip-flop, the two genes inhibiting each other as explained before (Fig D). If other forms of self-regulation are added to this basic structure the resulting structure still posses two different stable states, and such it still remains as bistable switch, albeit the solution of their dynamics are altered from the initial [17].

An analogous symmetry breaking process with the circuit generated from a Feed-Forward Fiber (FFF) building block (from Fig B) instead, shown on the bottom part of Fig DB, gives a circuit resembling a clocked *SR flip-flop* [5,22], which basically acts as a more complex *flip-flop* or toggle-switch. A FFF consists of a Feed-Forward Loop (FFL) motif (a three gene structure of genes X, Y and Z, where gene X regulates both Y and Z, and gene Y also regulating Z) [23] with an extra self-regulation on gene Y. This self-regulation in Y induces synchronization on both genes Y and Z, since it induces their input trees to be identical [5,11]. Depending on the signs of both regulations by X and Y, the inputs received by gene Z can be coherent or incoherent, classifying the FFLs as either coherent and incoherent [2,23]. Analogously, the FFFs are classified as SAT-FFF (satisfied) or UNSAT-FFF (unsatisfied) [5], respectively. UNSAT-FFF meaning that the two inputs are incoherent between each other and thus may produce an oscillating dynamic for the synchronized genes from their dynamical equations, given the competing and contradictory inputs [5], meaning this circuit works as a clock component.

This process can be continued with more complex fiber building block structures (from Fig B), leading to

a hierarchy of broken symmetry circuits as shown in Ref. [5], for example with Fibonacci fibers to give Fibonacci circuits, analogous to a JK flip-flop in electronics. However, these more complex circuits were not observed on the bacterial GRN studied in this work, but on more complex species, like yeast and human.

C.5 Step V: Identifying simple directed cycles

As discussed earlier, the SCCs can be thought of as "signal vortices" where the signals can cycle. These vortices are relevant because they are constituted of feedback loops (a form of cycle), without which the GRN's computational capacity would be drastically reduced to a "combinatoric" only nature. Indeed, feedback loops are what allow for the more complex dynamics of the logic circuits discussed above and why they are embedded in the SCCs. The SCCs, being these "signal vortices", in fact correspond to a very intricate cobweb of feedback loops.

After reducing a network via the k-core decomposition as described in section **Step II: Injective Fibrations**, all the remaining nodes have an output degree of at least one, i.e. all "null pathways" (pahtways ending at nodes with no-outputs) have been removed. This means that the SCCs are left intact during the k-core decomposition. This is important not only given that all the logic circuits belong to the SCCs but due to the interconnectedness of the nodes within a SCC, different circuits on a same SCC will therefore be intertwined with each other in non-trivial ways. In a sense, the SCCs consist of a very complicated entanglement of logic circuits.

In order to study the interconnectedness and interplay between these circuits, as well as to understand the structure of the SCCs we take a look at the independent simple cycles present in the minimal GRN. Such cycles are important not only because they connect different logic circuits and give structure to the SCCs but because, in itself, a cycle represents a form of longer-term memory, as a messaging signal is looping around the cycle.

For this, we look for all the independent simple directed cycles in the network, where a simple directed cycle is a closed path that crosses each node just once, except for the initial/final node. We search for a list of independent simple cycles, the cycle base, as all other cycles can be constructed by a sum of these, given that they span the cycle vector space [24, 25]. This is related to the concept of Betti Numbers in simplicial homology [26], where the k-th Betti number illustrates the number of k-dimensional holes in a space. In this sense, a cycle describes a type of topological hole. In particular, in the case of undirected graphs, the first Betti number $b_0 = |CC|$ where $|CC|$ corresponds to the number of connected components, while the next Betti number $b_1 = |E| - |N| - |CC|$ gives the number of independent simple cycles [27].

However, in order to construct this cycle base, cycles must be allowed to traverse a directed edge in the opposite direction (by having a negative contribution). For example, in a feed-forward loop motif it is possible to construct a cycle if one traverses at least one of the edges in the opposite, "negative", direction [24]. However, this does not make biological sense, and so we work with an "incomplete" base of cycles only allowed by existing pathways in the network, whose combinations span only the biologically existing pathways in the network. Under this mathematical restriction, in directed graphs, determining the number of cycles is a NP-complete problem [25]. See SI for more details on the algorithm for finding the cycles.

Since a self-loop or buckle just connects a node to itself in a cycle of length 1, they don't really contribute to the computational machinery and hence, as throughout, we ignore them for our cycles analysis.

D Details of the Gene Regulatory Network of *E. coli*

Details on signal vortices In Fig F we can see the fibers regulated by all the SCCs and their corresponding bases. Perhaps unsurprisingly, we see that *crp-fis* regulates the biggest fibers. Also of interest is that there are some fibers that are jointly regulated by two SCCs and that *uxuR-exuR* and *galR-galS* do not regulate any fiber of their own although they do regulate other genes and operons, just not synchronously.

The importance of the carbon *crp-fis* SCC is exemplified in the minimal network shown as the result of the

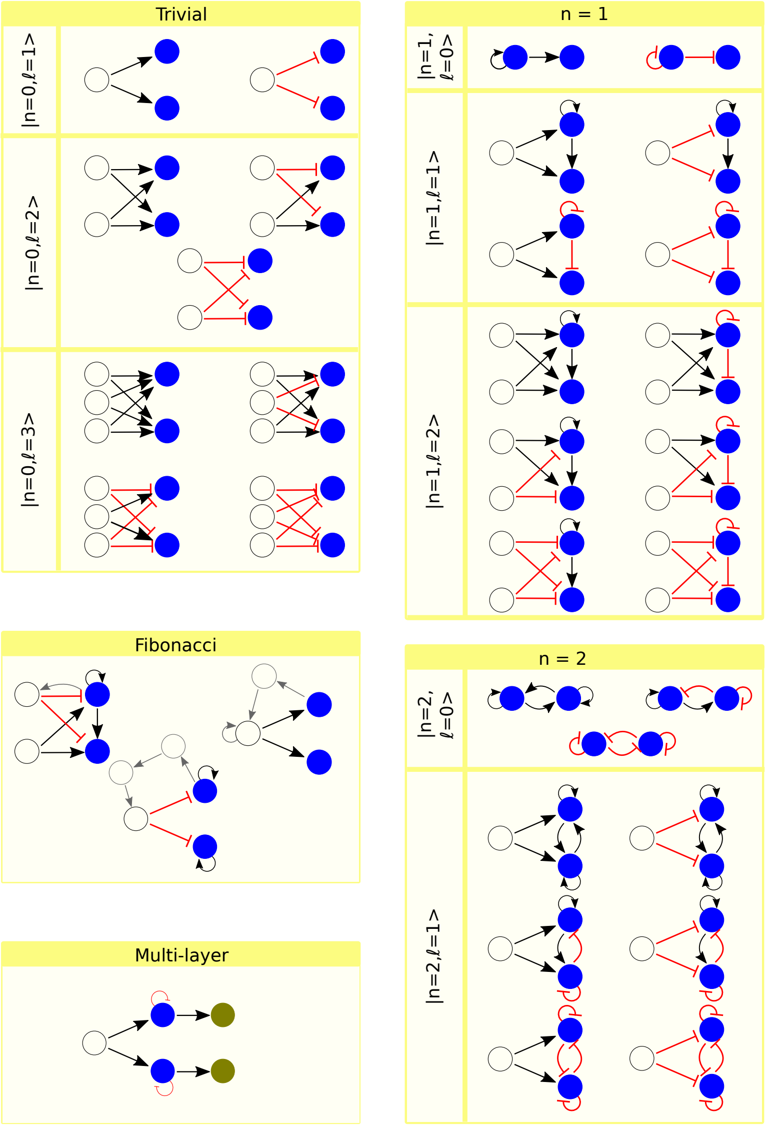

Fig. E. Possible circuits of the observed building block structures from *E. coli* and *B. subtilis*. We take the observed $|n, \ell\rangle$ classes in both bacteria and show all the possible combinations of activation and inhibition regulations for each class, i.e. all possible configurations which would still have a symmetric pair of nodes. In order for symmetry to occur the pair of synchronous nodes must receive an identical input tree. For the $n = 1$ cases, this can occur by a node with a self loop also regulating the other node in the fiber as shown, or alternatively by both nodes having a self-loop.

reduction process. When considered as an effective network, where each SCC is a super-node, the minimal GRN is a tree structure between the SCCs, i.e. without cycles and feed-forward. At the root of the tree is the carbon SCC which works as a type of master regulator, controlling the rest of the SCCs. All of these SCCs are regulated by different genes. For example, *cra*-fiber and *ihfAB* regulate the carbon SCC. The *galR-galS* and the *uxuR-exuR* SCCs receive signals from *crp-fis*, but do not receive or send signals to the rest of the SCCs. They compute their state solely based on the input of *crp-fis* SCC and then send their corresponding outputs to the genes that they regulate.

The other SCCs are arranged in the shape of a feed-forward motif: *crp-fis* SCC feeding the *soxS* SCC and *pH* SCC, the *pH* SCC also receives regulation from the *soxS* SCC (carbon → ph; carbon → stress; stress → ph) and another similar structure, with *marA-rob* SCC instead of *pH* SCC: being regulated from both *crp-fis*

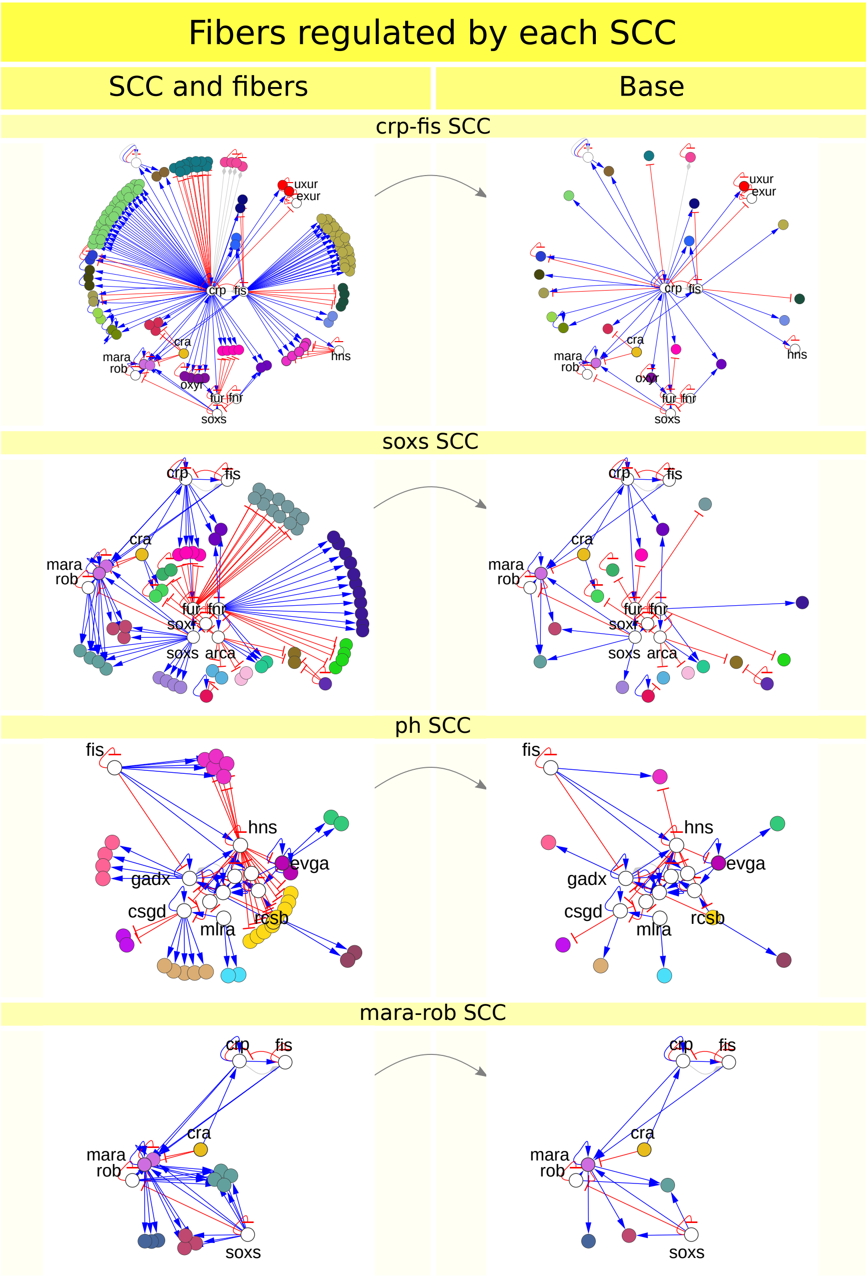

Fig. F. Example of a symmetry fibration applied to the SCCs and the fibers they regulate in *E. coli*'s operon GRN. On the left, genes that belong to the same fiber share the same color. On the right all the fibers have been collapsed to a single representative node per fiber. Nodes shown with labels are nodes belonging SCCs or regulators to them. It is interesting to note that quite a lot these fibers are actually regulated by not just the nodes of one SCC but by two SCCs. For example, in the second row for the *soxS* SCC there can be seen two fibers between this SCC and the *mara-rob* SCC in greenish and brownish colors respectively. The same thing occurs with two other fibers shared with the *crp-fis* and *soxS* SCCs in pinkish and purple colors. Out of the 6 SCC, the *galR-galS* and *uxur-exur* SCCs do not regulate any fibers of their own and thus do not appear on this figure. Blue edges with arrows represent activation, red edges with bars represent inhibition and grey edges with rhombuses represent dual regulation

and *soxS*. The general structure of the computational minimal core of the *E. coli's* GRN corresponds to two *feed-forward* structures between the SCCs.

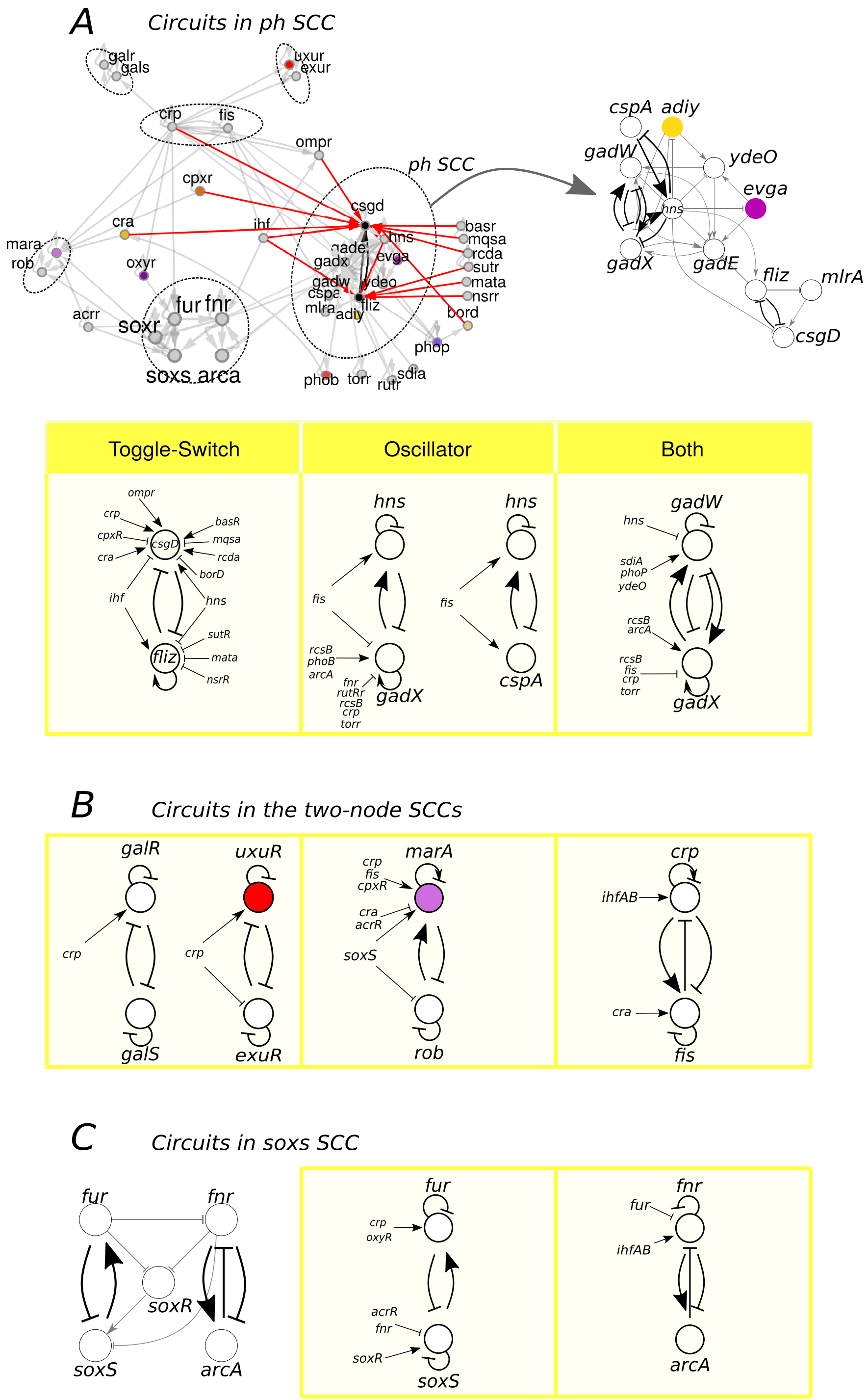

Fig. G. Circuits in the GRN of *E. coli*. **A** The minimal GRN of *E. coli* and the circuits embedded in it, shown with red links for the symmetry breaking inputs to the toggle-switch *fliz-csgd*. The biggest SCC is in charge mostly of pH responses. Colored nodes represent fibers. **B** The two-node SCCs and **C** the *soxS* SCC and its circuits. For each circuit, the incoming signals that break the symmetry are shown.

Details on gene circuits We found three circuits resembling toggle switches: each of them consists of two mutually repressed (MR) genes but with different self-regulations. Among them are the SCCs *galR-galS* and *uxuR-exuR*. For both of them, their only input gene is *crp*, which means that it could function as the logic *S-R* input selecting the state for the circuits. Both of these circuits present additional negative

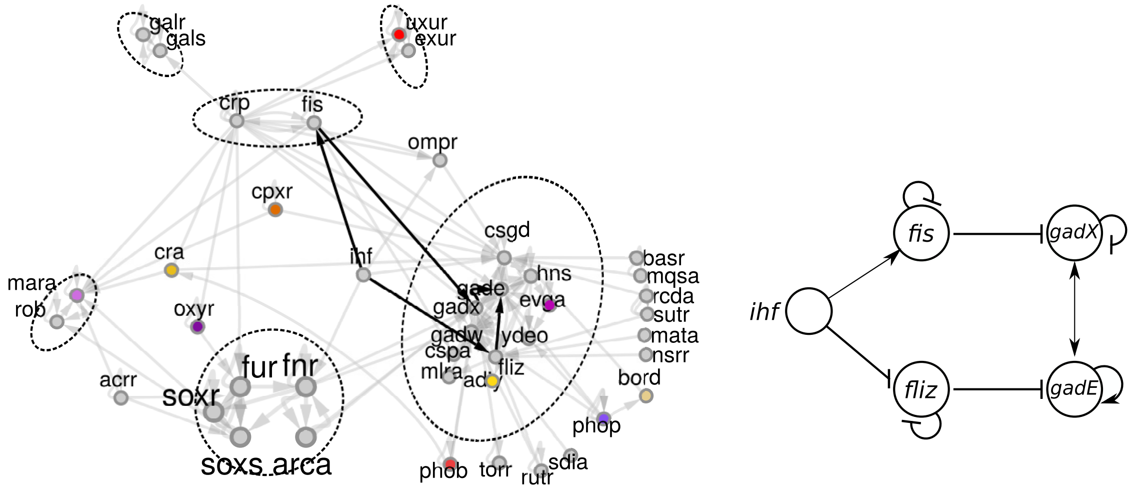

Fig. H. FFF circuit in *E. coli.* FFF circuits connecting the main SCC *crp-fis* to the *pH*-SCC. On the right we show the isolated circuit.

autoregulations in each gene (see Fig 5 in main text), so their dynamics requires further study, as compared to the classic toggle switch design.

In the case of *uxuR-exuR* cycle, with the regulations of *crp* this circuit then becomes the *Mutual Repression Network with Negative Autoregulation* studied by Hasan *et al.* [28]. This circuit can show two distinct stable states and may therefore serve as a memory: when *crp* is active, it can induce a state in which *uxuR* is expressed while *exuR* is repressed. The third possible toggle-switch-like circuit is between *csgD-fliz* in the *ph* SCC, shown in Fig G, with numerous possible ways for its symmetry breaking to occur, as can be seen in Fig GA. This circuit contains a positive autoregulation. As shown in [29], this allows for two stable states, making it possible for it to function as a memory device.

For NFBL (oscillator-type) circuits we observe 4 possible circuits: *rob* $\mapsto$ *marA* a SCC by itself; *soxS* $\mapsto$ *fur* in the *soxS* SCC; and *gadX* $\mapsto$ *hns* and *cspA* $\mapsto$ *hns* in the *ph* SCC. All of these are autoregulated, but the autoregulations in *gadX* $\mapsto$ *hns* (Fig G) in fact makes it a Smolen oscillator, the more robust type oscillator studied in Ref. [30].

There are also three pairs of nodes that can show various behaviors, since they can send various types of regulation message between them. For example, *crp* can send an activation or repression signal to *fis*, which means that *crp-fis* can be an MR circuit (toggle-switch type) or a NFBL circuit (an oscillating type), possibly even a Smolen oscillator given its autoregulations. Similarly, for *fnr-arcA* in the *soxS* SCC, which can be either. Lastly *gadW-gadX* in the *ph* SCC, in which both genes send both activating and repressing signals, can be a MR, a NFBL or a PAR feedback loop such as in a "lock-on" circuit; on top of this, one of the possible NFBL configurations includes a Smolen oscillator.

More surprisingly, we found FFF circuits, shown in Fig H, that connect the *pH* SCC to the master regulator *crp-fis* SCC and, through two different paths, to the *soxS* SCC, remarkably, the three FFFs are regulated by the same clock: *ihfAB*. It should be noted that for the three FFF circuits, the underlying feedback loop is a double positive autoregulation (PAR) feedback loop, which works as a bistable *lock-on* circuit; however, the circuits actually inhibit PAR.

E Testing the significance of network structures by comparison to randomized networks

In order to be biologically meaningful, the network structures found with ComSym should be statistically significant compared to the results obtained from randomized graphs. To test this, we created an ensemble

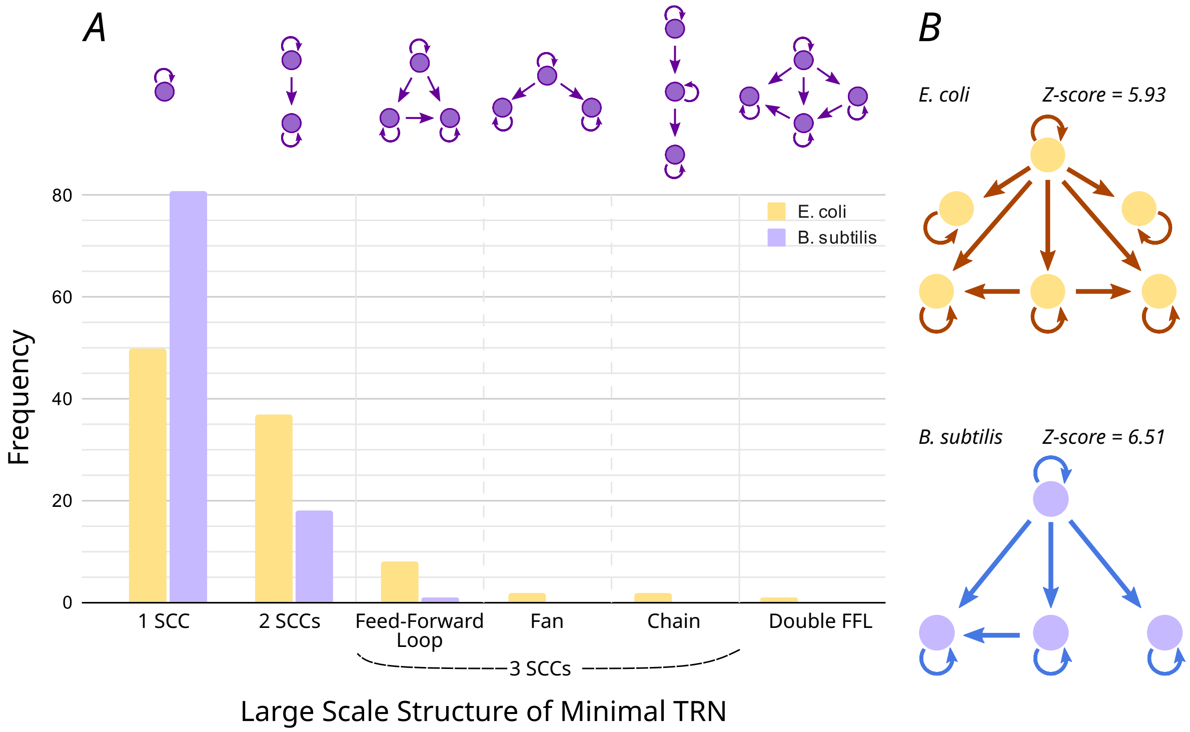

Fig. I. Statistically significant large-scale structures in *E. coli* and *B. subtilis* core GRNs. The structure between SCCs is compared to the corresponding structure in randomized networks with the same in- and out-degrees of all nodes (and preserving the edge types). **A** Histogram illustrating the distribution of the observed structures in the core of the random networks along with a sketch of the structure itself atop the histogram. Purple circles represent SCCs and arrows stand for edges between SCCs. **B** Structures observed in *E. coli*'s and *B. subtilis*'s core shown with the *Z-scores* of obtaining a structure with such number of SCCs from the randomized networks.

of 100 random networks, one corresponding to the characteristics of the *E. coli* network, and another for *B. subtilis*.

E.1 Procedure

Random networks were generated through a configuration model [31], that is, a random null model that preserves the in and out degrees of all network nodes, along with the type of edges, while individual connections between node pairs are randomized. In essence, we rewire the original network to randomize its edges, without adding new edges or nodes, at the end, the degree (both *in* and *out*) of each node remains unchanged. This is done by repeatedly choosing two edges from the original network at random and flipping their target nodes (i.e., edges $A \mapsto B$ and $X \mapsto Y$ become $A \mapsto Y$ and $X \mapsto B$), doing so repeatedly a number of times (10000 in our case). Self-edges are not included in the flipping. We then perform the same analysis on these random networks to compare the average structures across both ensembles to what was found on the *E. coli* and *B. subtilis* networks, respectively. The significance of a given property (e.g the number of appearances of a specific structure) is measured by its Z score from the mean values obtained from each ensemble; see Table A for the full breakdown.

E.2 Results

Our analysis reveals rich structures in the core of both bacteria's networks. Not only is the number of SCCs significant (with a Z-score > 5 for both bacterial networks), but also the connections between the SCCs

	E. coli			*B. subtilis*		
	N_{real}	$N_{\text{rand}} \pm$ SD	$Z - score$	N_{real}	$N_{\text{rand}} \pm$ SD	$Z - score$
**SCCs**						
Number of SCCs	6	1.61 ± 0.74	5.93	4	1.2 ± 0.43	6.51
Average SCC size	4	10.03 ± 7.94	-0.76	4.75	25 ± 11.7	-1.73
**Circuits**						
Total number of circuits	13	2.02 ± 1.39	7.9	8	3.95 ± 1.98	2.05
Toggle-switch	3	0.37 ± 0.58	4.53	2	-	Inf
Toggle-switch/Osc	2	0.13 ± 0.34	5.5	0	0.6 ± 0.94	-0.64
Oscillator	5	0.7 ± 0.77	5.58	2	2.97 ± 1.86	-0.52
Lock-on	0	0.45 ± 0.64	-0.7	4	—	Inf
Lock-on/Osc	0	0.17 ± 0.38	-0.45	—	—	—-
FFF	3	0.2 ± 0.6	4.67	0	0.38 ± 0.69	-0.55
**Cycles**						
Number of Cycles	41	40.72 ± 70.9	0.00	48	2037.84 ± 5560	-0.36
Average Cycle Length	3.68	8.35 ± 3.53	-1.32	4.88	15.49 ± 4.43	-2.4

Table A. Statistical significant structures in both GRNs. 100 random networks with identical in- and out-degrees of all nodes and identical edge types were generated, for both of *E. coli*'s and *B. subtilis*'s distributions. From these randomly generated networks we calculated the average and standard deviation quantities ($N_{rand} \pm SD$) to compare with the observed ones in the real distribution. Z-scores of each quantity were calculated to show the statistical significance of the findings.

form an interesting structure for both bacteria. We can see this structure for *E. coli* and for *B. subtilis* in Fig IB. In both networks, one *"central"* SCC regulates all the other SCCs in a *feed-forward* structure. We see two of these feed-forward structures in *E. coli* and one in *B. subtilis*, all involving the *central* SCC as the source. Most of the randomized networks, for both types of bacteria, have only one or two SCCs in their core, that is, basically no structure at all (see Fig I). Only 12 of the randomized *E. coli* networks show more than 2 SCCs, and only one of them shows 4 SCCs, while the ensemble of randomized *B. subtilis* networks contains only one network with 3 SCCs.

The average SCC size is not highly significant (according to its Z-score alone). We observe that the *size* of the SCCs decreases as the *number* of the components increases, which means that the more components found in these random networks, the smaller they were. For both *E. coli*'s and *B. subtilis*' GRNs, however, we found as many as 6 and 4 SCCs, some with sizes of 11 and 13 nodes, respectively. This is in sharp contrast to any structure observed in the randomized networks. For *E. coli*, the average size of the SCC, if the *only one* component is present, is 16.76 however, if there is *more than one* SCC, the average size now becomes 6.79; the biggest component has a size of 31 nodes for random networks with it being the only one for that particular network, on the other hand for random networks with three components its only 18 nodes in size. The same trend is true for *B. subtilis.*

Aside from this large-scale structure, we also found a variety of logic circuits on the smaller scale, with 12 circuits in total (Z-score = 7.9) for *E. coli* and 8 circuits (Z-score = 2.05) for *B. subtilis.* The appearance of circuits is very specific. In the case of *E. coli*, the randomized networks show no preference towards any particular type of circuit: the expected count numbers for all types of circuits are lower than 1. In the real network the count numbers of the observed circuit types were much higher, with significant Z-scores, see Table A. In the *B. subtilis* random networks, there is a bias towards the presence of oscillators of which we found 2 in the real network. We also found 2 toggle-switches and 4 lock-ons, although none of the two were found in the randomized networks.

F Algorithms and pseudocode

F.1 Minimal Balanced Coloring algorithm

This process of partitioning a network into sets of fibers with isomorphic input tress is equivalent to finding the minimal balanced coloring or balanced equivalence relations of the network [32,33]. A balanced coloring of a network occurs when the colors assigned on the nodes are such that they are balanced, which is to say that nodes in a given color receive the same number of inputs from each color they receive. This corresponds to an equivalence relation on the set of nodes [34], partitioning the network into balanced equivalence relations, hence its name [32,33]. It is worth mentioning that undirected networks can have multiple balanced colorings including some exotic ones [35,36], however, for the case of directed networks, given that the order of signals is more clearly defined, this does not seem to be an issue.

Our algorithm is based on a minimal balanced coloring algorithm proposed by Aldis and Kamei [11,32–34]. The algorithm starts by giving all the nodes the same color, except for the nodes with no input, since biologically there is no reason for them to be synchronous. Then the color of the nodes are recalculated: nodes are given the same color if they receive the same amount of inputs for every color they receive. For example, two genes that both receive one blue and one red input (just to say some colors as examples) are given a same color. If a third node receives two blue inputs and one red input, it is given a different color. By changing the colors, the color inputs of the nodes change as well. The colors are then re-calculated again based on the new color-input relations. Once the coloring is stable, the program is stopped. At this point, we have found the coloring partition where each color is a fiber. The code outputs the minimal balanced coloring, which represents the maximal fibration symmetry of the graph. This means that there are other coloring partitions that are not minimal balanced, for instance, we can always break a single fiber into two different colors, and decrease the symmetry, or equivalently, increase the number of colors, and this new coloring will not be the minimal. See [11] and its Supplementary Information for more information, as well as [19]. Our implementation of the Minimal Balanced Coloring algorithm can be found at `https://github.com/makselab/MinimalTRNCodes`.

One inadvertent advantage of these biological networks that show fibration symmetries and not automorphisms is the computational cost of calculating them. The *graph isomorphism problem* attempts to determine whether two finite graphs are isomorphic. It is a very active field of research as it is an immensely complicated problem to solve generally, Babai [37] has shown that graph isomorphisms can be solved in quasi-polynomial time; however, no polynomial-time algorithms are known today. It is still not clear if Graph Isomorphisms belongs to NP-complete [37]. Fortunately for us, however, since graph automorphisms are too restrictive for these real biological networks we only need to look on input tree isomorphisms instead, which are much less computationally costly: even for a pair of infinite input trees, it suffices to show the isomorphism up to $N_G - 1$ layers of the trees to determine the isomorphism [38]. Furthermore, the balanced coloring algorithm scales only with polynomial time [32–34].

F.2 ComSym Method

The entire code can be found at `https://github.com/luisalvarez96/MinimalTRN`.

Steps I to III Algorithm 1 shows Steps I to III. Although the column $\mathbf{N}[\textit{"FiberSize"}]$ is not directly used in the above pseudocode, it is a necessary output for the code in order to separate the nodes that belong to a fiber from those that do not. This is because after **Step I**, having collapsed all fibers into a single node, there are no repeated values in $\mathbf{N}_{col}[\textit{"FiberId"}]$, and hence without having the column $\mathbf{N}_{col}[\textit{"FiberSize"}]$ it would be impossible to tell which nodes belong to a fiber. This is because the *Minimal Balanced Coloring* algorithm returns a *"FiberId"* value *for each* node, even for nodes with no fiber (or single-fiber nodes).

With the output of this code, we can group nodes according to $\mathbf{N}_{min}[\textit{"SCCId"}]$, to determine the nodes that belong to a SCC bigger than one, determine how many SCCs are there, and study the interaction between them to finally reveal the *large-scale structure* of the minimal network.

Algorithm 1 ComSym Method: Steps I-III.

Notation:

- **N**[*"Label", "FiberId"*] referes to a list, or data frame, **N** with columns *"Label"*, and *"FiberId"*.
- **N**[*"Label"*] refers to column *"Label"* of data frame **N**.

Input: A list of edges **E**[*"Source", "Target", "Type"(optional)*], from graph $G = (N, E)$, where columns stand for: source node label, target node label, and type of interaction (activation, inhibition, etc.).
Output: A list $\mathbf{N}_{min}$ of nodes and a list $\mathbf{E}_{min}$ of edges (forming the minimal graph $G_{min} = (N_{min}, E_{min})$ of graph G).

Obtaining the Fibers (colors):

N[*"Label", "FiberId"*] ← *Minimal Balanced Coloring*(**E**) (see Section F.1 and SI from [11]).
Each row in **N** corresponds to a gene, with columns *Label*: gene name for each node; and *FiberId*: the assigned fiber (or color) for the corresponding node.
Add column *FiberSize* (size of the corresponding node's fiber) to **N**: **N**[*"Label", "FiberId", "FiberSize"*].

Step I: Collapsing (symmetry fibration)

Initialize an empty data frame $\mathbf{N}_{col}$ (for collapsed nodes).
for each fiber in **N**[*"FiberId"*] **do**
    Select one node (row) in **N** from that fiber and append its entire row to $\mathbf{N}_{col}$.
$\mathbf{E}_{col}$ ← From **E** select rows **if E**[*"Target"*] in $\mathbf{N}_{col}$[*"Label"*] (for collapsed edges).
for each edge in $\mathbf{E}_{col}$ **do**
    if $\mathbf{E}_{col}$[*"Source"*] not in $\mathbf{N}_{col}$[*"Label"*] **then**:
        Find the row in $\mathbf{N}_{col}$ where $\mathbf{N}_{col}$[*"FiberId"*] == *FiberId* from $\mathbf{E}_{col}$[*"Source"*].
        Replace $\mathbf{E}_{col}$[*"Source"*] with $\mathbf{N}_{col}$[*"Label"*] from the found row.

Step II: Pruning (k_{out}-core decomposition)

Add column *Outdegree* (outdegree for the corresponding gene, self-loops not included) to $\mathbf{N}_{col}$: $\mathbf{N}_{col}$[*"Label", "FiberId", "FiberSize", "Outdegree"*]
$\mathbf{N}_{min}$ ← From $\mathbf{N}_{col}$ select rows **if** $\mathbf{N}_{col}$[*"Outdegree"*] > 0
$\mathbf{E}_{min}$ ← From $\mathbf{E}_{col}$ select rows **if** $\mathbf{E}_{col}$[*"Target"*] in $\mathbf{N}_{min}$[*"Label"*]
while number of rows of $\mathbf{N}_{min}$ is not equal to number of rows of $\mathbf{N}_{col}$ **do**:
    Recalculate $\mathbf{N}_{min}$[*"Outdegree"*]
    $\mathbf{N}_{col}$ ← $\mathbf{N}_{min}$
    $\mathbf{E}_{col}$ ← $\mathbf{E}_{min}$
    $\mathbf{N}_{min}$ ← From $\mathbf{N}_{min}$ select rows **if** $\mathbf{N}_{min}$[*"Outdegree"*] > 0
    $\mathbf{E}_{min}$ ← From $\mathbf{E}_{min}$ select rows **if** $\mathbf{E}_{min}$[*"Target"*] in $\mathbf{N}_{min}$[*"Label"*]

Step III. Large-scale structure of the minimal network (SCCs)

Add column *SCCId* (numerical identifier for the SCC the corresponding node belongs to) to $\mathbf{N}_{min}$: $\mathbf{N}_{min}$[*"Label", "FiberId", "FiberSize", "Outdegree", "SCCId"*]

return $\mathbf{N}_{min}$[*"Label", "FiberId", "FiberSize", "Outdegree", "SCCId"*]
and $\mathbf{E}_{min}$[*"Source", "Target", "Type"*]

Step IV To find the logic circuits, we run a modified version of the algorithm developed by Leifer *et al.* in Ref. [5] looking for the induced subgraphs of the network whose connectivity is identical to the logic circuits we are looking for. The algorithm consists of essentially two steps.

Algorithm 2 ComSym Method: Steps IV.

Notation: same as in previous Algorithm 1
Input: A list of edges $\mathbf{E}_{min}$[*"Source", "Target", "Type"(optional)*].
Initialize: An adjacency matrix **Adj** for each circuit to look for.
Output: A list **C** of circuits.

```
E ← From E select rows if E["Source"] is not equal to E["Target"]
E ← From E remove duplicated rows
C ← search for subgraphs of E_min isomorphic to Adj
C ← From C select only induced subgraphs
return C
```

The **Adj** matrices for the circuits correspond to:

$$AR = \begin{bmatrix} 0 & 1 \\ 1 & 0 \end{bmatrix} \quad FFF = \begin{bmatrix} 0 & 0 & 0 & 0 & 0 \\ 1 & 0 & 0 & 0 & 1 \\ 1 & 0 & 0 & 1 & 0 \\ 0 & 1 & 0 & 0 & 1 \\ 0 & 0 & 1 & 1 & 0 \end{bmatrix}$$

For circuits that originate from the duplication of an auto-regulated (AR) gene or a FeedForward fiber (FFF) building block, respectively. AR circuits can then be further classified into a toggle-switch, a lock-on circuit, or an oscillator depending on their type of edges. A toggle-switch corresponds to both edges being repressive, an oscillator to one negative and one positive and a lock-on for both edges as activations. In actuality, for both studied GRNs, the first two lines in Algorithm 2 are required since virtually no circuits are exactly identical to the "neat" and nicely behaving circuits design and implemented synthetically. Perhaps it should come as no surprise that actual in-vivo reality is more complicated. Some of the observed circuits, as a result, have a slightly different topology due to self-regulations of some genes in the circuits or due to multiplicity of "paralel" edges, so their specific dynamical behavior requires further study.

Step V Both networks studied here are small enough to be analyzed using an algorithm loosely based on *Johnson's algorithm* [39] and shown on Algorithm 3. It starts by breaking the network into SCCs and searches for cycles within each one. Each component is analyzed by 1) enumerating the nodes in the SCC, 2) choosing one node (the initial/final node of the cycle), 3) looking for the outgoing neighbors of this node, and 4) looking for all the simple paths (paths without repeating nodes) back from each neighbor to the initial/final node.

Algorithm 3 ComSym Method: Steps V.

Notation: same as in previous Algorithm 1
Input: $\mathbf{N}_{min}$[*"Label", "FiberId", "FiberSize", "Outdegree", "SCCId"*]
and $\mathbf{E}_{min}$[*"Source", "Target", "Type"(optional)*].
Output: A list **C** of cycles.

Identify all *SCCId*'s for the SCCs from $\mathbf{N}_{min}$[*"SCCId"*]
Initialize empty list *Cycles*
for every *SCCId* in SCCs **do**
    enumerate all nodes in *SCCId* (order is arbitrary, results will be the same regardless)
    NodeCount ← Number of nodes in list of enumerated nodes
    while *NodeCount* is not equal to 1 **do**
        node ← first node from list of enumerated nodes
        neighbors ← out-neighbors from node
        Initialize empty list *paths*
        for *neighbor* in *neighbors* **do**
            find all simple paths from *neighbor* to *node* in $\mathbf{E}_{min}$
            append paths from *neighbor* to *node* to *paths*
        Add columns *SCC* (*SCCId* of current SCC) and *Lenght* (length of each path) to *paths*
        Append *paths* to *Cycles*
        Remove *node* from list of enumerated nodes
        NodeCount ← Number of nodes in list of enumerated nodes
C ← From **C** remove duplicated rows
return C

References

1. Gardner T.S., Cantor C.R., and Collins J.J. Construction of a genetic toggle switch in *Escherichia coli*. *Nature*, 403(6767):339–342, 2000.

2. Alon U. *An introduction to systems biology: design principles of biological circuits*. CRC press, 2019.

3. Bintu L., Buchler N. E., Garcia H. G., Gerland U., Hwa T., Kondev J., and Phillips R. Transcriptional regulation by numbers: models. *Curr Opin Genet Dev*, 15:116-124, 2005.

4. Klipp E., Liebermeister W., Wierling C., and Kowald A. *Systems biology: a textbook*. John Wiley & Sons, 2016.

5. Leifer I., Morone F., Reis S.D., Andrade Jr J.S., Sigman M., and Makse H.A. Circuits with broken fibration symmetries perform core logic computations in biological networks. *PLoS computational biology*, 16(6):e1007776, 2020.

6. Oishi K., and Klavins E. Framework for engineering finite state machines in gene regulatory networks. *ACS synthetic biology*, 3(9):652–665, 2014.

7. Setty Y., Mayo A. E., Surette M. G. and Alon U. Detailed map of a cis-regulatory input function. *PNAS*, 100(13):7702-7707, 2003.

8. Kaplan S., Bren A., Zaslaver A., Dekel E. and Alon U. Diverse two-dimensional input functions control bacterial sugar genes. *Molecular Cell*, 29:786-792, 2008.

9. Mayo A. E., Setty Y., Shavit S., Zaslaver A., and Alon U. Plasticity of the cis-regulatory input function of a gene. *PLoS Biol.*, 4(4):e45, 2006.

10. Leifer I., Sánchez-Pérez M., Ishida C., and Makse H.A. Predicting synchronized gene coexpression patterns from fibration symmetries in gene regulatory networks in bacteria. *BMC bioinformatics*, 22(1):1–34, 2021.

11. Morone F., Leifer I., and Makse H.A. Fibration symmetries uncover the building blocks of biological networks. *Proceedings of the National Academy of Sciences*, 117(15):8306–8314, 2020.

12. Harary F. *Graph theory.* Addison-Wesley, 1993.

13. DeVille L., and Lerman E. Modular dynamical systems on networks. *J. Eur. Math. Soc.*, 17:2977–3013, 2015.

14. Morone F., and Makse H.A. Symmetry group factorization reveals the structure-function relation in the neural connectome of *Caenorhabditis elegans. Nature communications*, 10(1):1–13, 2019.

15. Golubitsky M., and Stewart I. Nonlinear dynamics of networks: the groupoid formalism. *Bulletin of the american mathematical society*, 43(3):305–364, 2006.

16. Stewart I., Golubitsky M., and Pivato M. Symmetry groupoids and patterns of synchrony in coupled cell networks. *SIAM Journal on Applied Dynamical Systems*, 2(4):609–646, 2003.

17. Stewart I., Reis S.D., and Makse H.A. Gene regulatory circuits as biological building blocks: Dynamics and bifurcations. *Journal of the Royal Society Interface*, 21(217):20240386 2024.

18. Boldi P., and Vigna S. Fibrations of graphs. *Discrete Mathematics*, 243(1-3):21–66, 2002.

19. Monteiro H.S., Leifer I., Reis S.D., Andrade Jr J.S., and Makse H.A. Fast algorithm to identify cluster synchrony through fibration symmetries in large information-processing networks. *Chaos*, 32(3):033120 2022.

20. Malliaros F.D., Giatsidis C., Papadopoulos A.N., and Vazirgiannis M. The core decomposition of networks: Theory, algorithms and applications. *The VLDB Journal*, 29(1):61–92, 2020.

21. Kitsak M., Gallos L.K., Havlin S., Liljeros F., Muchnik L., Stanley H.E., and Makse H.A. Identification of influential spreaders in complex networks. *Nature physics*, 6(11):888–893, 2010.

22. Tanenbaum A.S. *Structured computer organization.* Pearson Education India, 2016.

23. Mangan S., and Alon U. Structure and function of the feed-forward loop network motif. *Proceedings of the National Academy of Sciences*, 100(21):11980–11985, 2003.

24. Kavitha T., Liebchen C., Mehlhorn K., Michail D., Rizzi R., Ueckerdt T., and Zweig K.A. Cycle bases in graphs characterization, algorithms, complexity, and applications. *Computer Science Review*, 3(4):199–243, 2009.

25. Gruber H. Digraph complexity measures and applications in formal language theory. *Discrete Mathematics & Theoretical Computer Science*, 14, 2012.

26. Munkres J.R. *Elements of algebraic topology.* CRC press, 2018.

27. Berge C. *The theory of graphs.* Courier Corporation, 2001.

28. Hasan A.B., Kurata H., and Pechmann S. Improvement of the memory function of a mutual repression network in a stochastic environment by negative autoregulation. *BMC bioinformatics*, 20(1):1–14, 2019.

29. Leon M., Woods M.L., Fedorec A.J., and Barnes C.P. A computational method for the investigation of multistable systems and its application to genetic switches. *BMC systems biology*, 10(1):1–12, 2016.

30. Stricker J., Cookson S., Bennett M.R., Mather W.H., Tsimring L.S., and Hasty J. A fast, robust and tunable synthetic gene oscillator. *Nature*, 456(7221):516–519, 2008.

31. Molloy M., and Reed B. A critical point for random graphs with a given degree sequence. *Random structures & algorithms*, 6(2-3):161–180, 1995.

32. Aldis J.W. A polynomial time algorithm to determine maximal balanced equivalence relations. *International Journal of Bifurcation and Chaos*, 18(02):407–427, 2008.

33. Kamei H., and Cock P.J. Computation of balanced equivalence relations and their lattice for a coupled cell network. *SIAM Journal on Applied Dynamical Systems*, 12(1):352–382, 2013.

34. Cardon A., and Crochemore M. Partitioning a graph in O (|V|log2|V|). *Theoretical Computer Science*, 19(1):85–98, 1982.

35. Golubitsky M., Nicol M., and Stewart I. Some curious phenomena in coupled cell networks. *Journal of Nonlinear Science*, 14:207–236, 2004.

36. Golubitsky M., and Stewart I. Rigid patterns of synchrony for equilibria and periodic cycles in network dynamics. *Chaos: An Interdisciplinary Journal of Nonlinear Science*, 26(9), 2016.

37. Babai L. Graph isomorphism in quasipolynomial time. *Proceedings of the forty-eighth annual ACM symposium on Theory of Computing*, pages 684–697, 2016.

38. Norris N. Universal covers of graphs: isomorphism to depth n- 1 implies isomorphism to all depths. *Discrete Applied Mathematics*, 56(1):61–74, 1995.

39. Johnson D.B. Efficient algorithms for shortest paths in sparse networks. *Journal of the ACM (JACM)*, 24(1):1–13, 1977.
