# Supplementary material for: Complexity reduction by symmetry: Uncovering the minimal regulatory network for logical computation in bacteria
Source: PLoS Comput Biol. 2025 Apr 24;21(4):e1013005. doi: 10.1371/journal.pcbi.1013005 (PMC12048163; doi:10.1371/journal.pcbi.1013005)
Supplement: Attachment — Submitted filename: Minimal TRN reviews.pdf [file pcbi.1013005.s002.pdf]

Luis Álvarez García
Levich Institute and Department of Physics
140th St. and Convent Ave.
New York, NY 10031
City College of New York

Phone: (929) 507-3539

Email: luisalvarez.10.96@gmail.com

Dear William Cannon and Stacey Finley, February 7th, 2025
Academic Editor and Section Editor respectively
PLOS Computational Biology

Thank you very much for facilitating a review of the manuscript "Complexity reduction by symmetry: uncovering the minimal regulatory network for logical computation in bacteria". We would like to present for your consideration and the reviewers a significantly revised version of the manuscript. Below we present a point by point response to the reviewers comments as well as detail the changes done to the manuscript in response to the reviewers concerns.

The manuscript has been significantly reduced, from the submitted 53 pages to 30. A lot of repetition has been removed, some sections have been moved to a new SI and the overall structure and flow of the paper has been significantly edited with readability and clarity for the reader in mind, along with significant rewriting as well. Because of this, it is not possible to highlight all of the changes in the manuscript to keep track, although the new writing to address some of the reviewers' specific comments are highlighted accordingly.

Detail response to Reviewer # 1

We want to thank the reviewer for a very insightful and helpful review of the manuscript.

In this study, the authors develop a method to reduce the complexity of transcriptional regulatory networks based on relaxed principles of symmetry and k-core decomposition. The minimal TRN is decomposed into strongly connected components; symmetry breaking and simple cycles are sought to describe its most basic structures.

It was difficult for me to read and understand the study because it is a large manuscript—potentially excessively large—and readers can benefit from the most concrete writing.
The manuscript has been significantly reduced, from the submitted 53 pages to 30. A lot of repetition has been removed, some sections have been moved to a new SI and the overall structure and flow of the paper has been significantly edited with readability and clarity for the reader in mind.

Given my biological background, it is difficult to appreciate the potential utility of the generated knowledge and how this can contribute to advancing the explanation of how these model microorganisms process the environment's signal and are processed internally to respond adequately.

The work presented provides a systematic method to extract the minimal set of a network in charge of the "decision-making" process. This systematic approach in itself provides a significant contribution on how to understand these complicated systems and can be readily applied to other similar systems. By providing a transparent breakdown of the very complicated global structure of these systems, this work paves the way on how to identify the crucial components of the system that control their global dynamics and behavior.

Some sections, like antecedents, methods, and results, are mixed. Some suggestions to improve the manuscript are:

Lines 1409-1411 should be part of methods.
This is specific preprocessing for the B. subtilis data; it's not a required, general, methodological step. Since the Methods section is already lengthy enough, we decided to leave it in this section rather than create a new subsection in methods for preprocessing of each dataset. We feel this makes for better readability..
Lines 1481-1483 should be part of the methods. Moreover, add a description of how the random networks were created.
Since the actual procedure for obtaining randomized degree-preserved networks is commonly used to test statistical significance of observed structures within a network, it does not feel necessary to emphasize this procedure in the Methods section. Rather we are interested in

showing the results we obtain from this - hence we chose to leave it at the Results section.

However, in order to make sure it is clear how these randomized networks are created we added lines 635-638 for a conceptual explanation, and refer to Section 5.1 of the SI where it is explained in full detail.

When analyzing TRNs in E. coli and B. subtilis, it is suggested that the subtitles be more descriptive for each case; for example, there are two subtitles called "Cycles."
We have addressed this throughout the manuscript

Examples of applying concepts to solve problems in other areas can be irrelevant to the present work. They may contribute to a shorter manuscript, be avoided, or only mentioned succinctly. For instance, lines 29-32 and 21-25
Taking the reviewer's suggestion of making the Introduction more concise, we have removed these parts from the introduction and only left a summarized version of how the use of physics in symmetries has inspired us and informed our work in Section 2.1

Some figures, for example, 8, indicate the type of regulation (positive, negative, or dual), but the methodology does not describe whether the type of regulation is considered and, if so, how it is processed.
To address this we have added lines 325-328.

ComSym's algorithm development seems to be a compilation of already-described methods repeated throughout the entire process. Section 2.7 repeats the previous sections. For instance, lines 1003-1007 are the same as 224-234.
Thanks to the reviewer for pointing out the repetition. In favor of conciseness, only section 2.4 outlines the steps of the method, while only a brief introductory notion on the Introduction, lines 117-134.

Figures are referred to away from the figure inset; for example, Fig. 7 (page 33) is cited first in the introduction (page 7) and then in section 2.3 (page 18).
We have addressed this throughout the manuscript, with perhaps the exception of Fig. 2 which is refer to in several instances for being a basic notion used throughout.

In lines 1418-1420, what conclusions were obtained from analyzing the B. subtilis network?
This has been addressed in section 3.3, specifically lines 590-607 we believe might be helpful.

What conclusions are obtained when comparing the structures of the minimal TRN in both species? - B. subtilis has a more simple large-scale structure in the minimal TRN but they both exhibit a very rich structure when compared to random networks.
We try to address this throughout lines 590-629.

Section 3.3.1 can be interpreted as a relationship between the network size and the minimum network size. Is there support for that?
We do not claim that there is a specific a priori way of knowing the size of the minimal TRN from the total size, but we address what type of structure will be obtained in general in lines 377-396.

In terms of adaptation, what does the following paragraph mean?
Our analysis suggests that the two bacterial networks resulted from an evolutionary selection for specific functional structures. (Line 1538)
We have rewritten section 3.4 in such a way that we hope addresses this concern.

The general idea is that because the observed structures are not a product of chance, then there must've been some evolutionary pressure selecting for these traits. See specifically lines 642-644.

In line 1662, can authors hypothesize what led E. coli or B. subtilis to adopt the structure of their TRN?
We can not say exactly what “trait” or set of traits are selected by the specific structures observed, as opposed to, for example, slightly different structures producing slightly different, less beneficial traits. This is a very tough question to try to address, we would need to perhaps model the entire system and see how alterations on it could affect the dynamics and behavior.

But we can speculate as to *how* these structures might have arised: the process of gene duplication seems a very plausible candidate for generating this structures. See for instance lines 767-790 in the main text and also section 3.4 in the SI.

In the line "since the main function of the bacterium cell is to process sugars." A reference may be required. This has been added.

Ideally, the assignment of possible functions (memory, oscillator, and toggle-switch) in section 3.2.2 should be linked with the biological function.
We agree with the reviewer's insight, and ideally we would have preferred to address it. However, doing so is not a trivial task at all, it would require determining specifically what the effects of each gene are under a number of circumstances and how their interactions also might affect the behaviour. See for instance references 41 and 42 of the main text.

The authors should hypothesize the implications of considering E. coli as a computer. What is the biological logic of designing a minimal synthetic network with the principles presented in the work, and how do they work together? Section 3.2.3
While we agree that ideally, a more in-depth analyses of how the dynamics of each circuit is affected by the longer cycles would be needed to have a concrete answer, unfortunately this work itself would amount to another paper.

Closely related to this idea, how specific dynamics might be affected by some of the observed "real-life" structures observed, as opposed to the carefully implemented circuits from synthetic biology, the last author of this paper Hernan Makse is preparing another paper on this topic, see reference 17 from the SI, although is still in preparation.

Can an output be generated on line 1561 using a TRN and then propagated into a full TRN, observing the same results as the responses from the full TRN?
In principle yes but having specific datasets for specific conditions and how to compare them with the output of a modeling of our minimalTRN is not a trivial task unfortunately. Ideally, this would be one of the next steps to take.

The biological role of the primary genes involved in minimal TRN is not described.
To some extent we tried to describe, in a very general level, what the roles of the SCCs are, we do this based on the general roles that the nodes within them are known to perform. See paragraph starting at line 527.

Reviewer #2:

Thanks to the reviewer for all the insightful observation for improving this work.

This paper describes a method for simplifying transcription networks from being very complicated, and completely unintuitive, to being vastly simpler and actually understandable. This is an important step to simplification that has potential widespread application. The authors demonstrate their approach using an E. coli network and a B. subtilis network.

This work described in this paper clearly represents an impressive amount of effort and appears to be significant. However, I am not an expert in this subfield of work and so I had a hard time understanding how much improvement this approach yields over existing methods.

I have three major critiques of the work.

First, the paper is extremely long (at somewhere around 20,000 words) and very mathematical, with minimal connection to actual biology. These aspects make the paper very hard to read. In my case, I have a physics background and reasonably strong math skills, but I still struggled to maintain interest in the work after about the first 20 pages. This was less than halfway through the manuscript. I suggest that the authors shorten the manuscript dramatically, moving substantial amounts of content to online supplementary material.
The manuscript has been significantly reduced, from the submitted 53 pages to 30. A lot of repetition has been removed, some sections have been moved to a new SI and the overall structure and flow of the paper has been significantly edited with readability and clarity for the reader in mind.

Second, the manuscript **does not discuss the broader field of transcription network simplification**, which makes it hard to assess how much of an improvement these new methods provide over existing work. It has many references to closely related work, but even

those papers are not described here. Most readers are not going to track down each of these references to gain this background material.
We mention specific relevant work into the structure of E. coli's TRN, as well as their limitations, in lines 57-72 for example, as well as 706-711, as well as the paragraph at line 751. In terms of a systematic reduction of a full genome, however, the closest efforts are something as done in reference 51 of the main text and associated literature. However, their approach is very different, by focusing on how the genome can be reduced by knocking *individual* genes and determining the *essential* genes that when knocked down the cell is not able to perform necessary functions. There is overlap and certainly it is very interesting to see how our results compare to theirs and how our results could maybe help explain some of theirs. However this would amount to another work in and of itself.

Third, the approximations that are made in this work appear to be large enough that I question if the final results adequately represent real biology. For example, symmetries are powerful in physics because they arise from underlying symmetries in nature. However, this does not appear to be the case in biology; instead, the symmetries identified here appear to arise primarily from the approximations that were made to the systems rather than from the properties of the systems themselves. Thus, I question if the symmetries identified here represent anything that's particularly interesting, or if they are just an applied method for simplifying transcriptional networks. As a related issue, this work appears to build on Boolean approximations for transcriptional networks, which have been known for a long time to be extremely coarse and not particularly accurate.
Thanks to the reviewer for a very insightful comment. We address this in lines 260-286, as well as lines 97-100. Certainly this is case where there is a considerable amount of idealization but we can point out to the fact that fibrations and fibers do actually predict well gene coexpression patterns, see previous work in reference 11 of main text. In relation to this, if the fibers are not drastically "destroyed" by the assumptions not being met, and instead being correlation levels, such as coexpression levels, the methodology for finding the *core* of the network in charge of the outward spread and control of dynamics is still very much valid. This is what I tried to explain in lines 278-286.
In terms of boolean approximations, indeed they are known to be very coarse, however, at the sort of conceptual level of determining an apparatus capable of organically carry out computations, it is relevant to point out to reference 21 of the main text. Particularly, what I point out to in lines 98-100.

As a minor issue, this paper has numerous spelling mistakes. I suggest doing a careful proofread and also running it through a spell-checker.
We hope to have addressed all such mistakes.

Reviewed by Steve Andrews

Reviewer #3:

A thank you to the reviewer for dedicating the time to provide useful feedback for improving this work.

This manuscript presents an approach for reducing ODE based networks to a minimal version that is referred to as the k-core of the network. The k-core is composed of a collection of strongly connected components. The method is based on collapsing nodes that belong to the same equivalence class using symmetry fibrations and by k-core decomposition. The reduced version of the network preserves the dynamics of the original network. The authors applied their reduction approach to two networks from bacteria (e. coli and b. subtilis).

I find this manuscript mostly well-written, and their approach could be useful to analyze other networks. However, some details of the method are missing and there are some inconsistencies in the name of the method. Therefore, I suggest the following revisions before recommending it for publication.

Major revisions:
1. The method is presented as a list of 5 steps. It'd be better to provide a pseudo-code of the reduction algorithm, so others can implement it.
This is a great suggestion, thank you very much. We have implemented this in section 6.2 of the SI.
2. Is the method called "ComSym" or "CoReSym"? In Section 2.7 it is presented as ComSym and later as CoReSym.
Thank you for pointing out this inconsistency. We have moved on to consistently call it ComSym.

Minor revisions:
1. The panel labels (a) and (b) in Fig. 1 are missing. We refer to the *left* panel and *right* panel.
2. On line 553, page 14, it says that "nodes 1 and 4 have no inputs", but node 1 has input from node 2, right? It is even shown in Fig. 3B. Thank you very much for pointing this out. Node 1 has an input indeed, this was a typo.
3. Reference [38] is a preprint from 2013. Has this been published? If so, please update this reference. Reference has been updated, reference 23 of the revised manuscript.
4. Change "fibation" to fibration on line 865. Thank you for pointing out this mistake!
5. Check formatting of reference [31]. Addressed
6. In the caption of Fig. 7, add a space before "Step two removes …" Addressed
